# Compatibility of *Isaria fumosorosea* (Hypocreales: Cordycipitaceae) Blastospores with Agricultural Chemicals Used for Management of the Asian Citrus Psyllid, *Diaphorina citri* (Hemiptera: Liviidae)

**DOI:** 10.3390/insects4040694

**Published:** 2013-11-26

**Authors:** Pasco B. Avery, David A. Pick, Luis F. Aristizábal, James Kerrigan, Charles A. Powell, Michael E. Rogers, Steven P. Arthurs

**Affiliations:** 1 Indian River Research and Education Center, University of Florida, Institute of Food and Agricultural Sciences, 2199 South Rock Road, Fort Pierce, FL 34945, USA; E-Mail: capowell@ufl.edu; 2 Mid-Florida Research and Education Center, Department of Entomology and Nematology, University of Florida, Institute of Food and Agricultural Sciences, 2725 Binion Road, Apopka, FL 32703, USA; E-Mails: pickdavid1@hotmail.com (D.A.P.); larist@ufl.edu (L.F.A.); james.k13@live.com (J.K.); spa@ufl.edu (S.P.A.); 3 Citrus Research and Education Center, Department of Entomology and Nematology, University of Florida, Institute of Food and Agricultural Sciences, 700 Experiment Station Road, Lake Alfred, FL 33850, USA; E-Mail: mrgrs@ufl.edu (M.E.R.)

**Keywords:** entomopathogenic fungi, citrus, pathogenicity, agrochemicals, bioassay, oils, fungicides, virulence, IPM

## Abstract

Biorational insecticides are being increasingly emphasized for inclusion in integrated pest management programs for invasive insects. The entomopathogenic fungus, *Isaria fumosorosea*, can be used to help manage the Asian citrus psyllid with minimal impact on beneficial arthropods, but its effectiveness may be compromised by agrochemicals used to control concurrent arthropod pests and diseases. We evaluated the compatibility of *I. fumosorosea* blastospores with a range of spray oils and copper-based fungicides registered for use in citrus groves. Results of laboratory and greenhouse tests showed a range of responses of the fungus to the different materials, including compatibility and incompatibility. Overall, *I. fumosorosea* growth *in vitro* was reduced least by petroleum-based materials and most by botanical oils and borax, and some of the copper-based fungicides, suggesting that tank mixing of *I. fumosorosea* with these latter products should be avoided. However, equivalent negative effects of test materials on fungal pathogenicity were not always observed in tests with adult psyllids. We hypothesize that some oils enhanced adherence of blastospores to the insect cuticle, overcoming negative impacts on germination. Our data show that care should be taken in selecting appropriate agrochemicals for tank-mixing with commercial formulations of entomopathogenic fungi for management of citrus pests. The prospects of using *I. fumosorosea* for managing the invasive Asian citrus psyllid and other citrus pests are discussed.

## 1. Introduction

The development of entomopathogenic fungi as mycopesticides against soft bodied insects and mites has a long history [1]. Mycopesticides have arguably seen a renaissance in recent years in some parts of the world, due to the limited effectiveness of insecticide programs against pests including thrips, whiteflies and mosquitoes, and issues of insecticide tolerant populations [2,3,4,5]. However, since entomopathogenic fungi often do not provide adequate control by themselves or against an entire pest complex, including diseases and weeds [6,7], in many cases they will need to be used in conjunction with other pesticides. Also, since entomopathogenic fungi require direct contact with the host, they may be mixed with a variety of adjuvants to enhance coverage and residual activity at the target site. 

A case in point, the Asian citrus psyllid, *Diaphorina citri* Kuwayama (Hemiptera: Liviidae), is a major pest in cultivated citrus due to its role in transmission of the ‘*Candidatus* Liberibacter’ species that cause citrus greening disease (huanglongbing, HLB) [8,9]. Since there is no cure for HLB, current control recommendations in Florida and other regions of the world include the frequent use of insecticides to control the vector [10,11]. Although insecticides from several chemical classes are considered effective at reducing psyllid populations [12], research indicates that even intensive insecticide programs are ineffective at preventing the spread of HLB [9,13]. An excessive reliance on a limited number of plant protection products has also resulted in reduced susceptibility of *D. citri* to insecticides including neonicotinoids, pyrethroids, organophosphates and carbamates [14,15]. Moreover, the current insecticide strategy is disruptive to the various beneficial species that are known to control pest outbreaks in the citrus groves, including exotic parasitoids released for classical biocontrol of *D. citri* in Florida and other regions [16,17].

A number of entomopathogenic fungi are known to naturally infect *D. citri* worldwide [18,19,20,21,22,23,24,25,26]. Amongst them, *Isaria fumosorosea* Wize (= *Paecilomyces fumosoroseus*) (Hypocreales: Cordycipitaceae) has received interest for use against *D. citri* in Florida [27,28,29,30]. Commercial blastospore formulations of *I. fumosorosea* are now registered in North America for use against pests of food crops [31]. However, a wide range of agrochemicals that are routinely applied in Florida citrus groves may impact the use of this fungus to target the psyllid. In this paper, we evaluated the compatibility of *I. fumosorosea* blastospores with a range of spray oils and copper-based and other fungicides registered for use in citrus groves.

## 2. Experimental Section

*Source of fungus, oils and insects:* A blastospore formulation of *I. fumosorosea* Apopka 97 strain (PFR-97 20% WDG, Certis USA, Columbia, MD) was used in our studies. Tested products included emulsified petroleum-based oils, citrus-derived alcohol ethoxylates, a silicone surfactant, and fungicides based on borax (sodium tetraborohydrate decahydrate) and copper (Table 1). 

**Table 1:** List of products, types, pesticide rates and categories used in bioassays.

Product/Manufacturer	Type^1^	Rate (% v/v) / acre	Use^2^
SuffOil-X™, BioWorks Inc. Victor, NY	P	1–2	I,M,F
Citrus Wrap™, Loveland Products, Inc., Greeley, CO	P	1–5 quarts	A
Griffin 435 Oil™, Ben Hill Griffin, Inc., Frostproof, FL	P	5–10 gals	I,M,F
PureSpray Green™, Whitmire Research Inc., St Louis, MO	P	0.75–2	I,M,F
Year-Round™, Summit, Baltimore, MD	P	1–2.9	I,M
Sylgard^®^ 309, Wilbur-Ellis, Fresno, CA	S	0.03–0.4	A
Neem Oil, Dyna-Gro, Richmond, CA	B	0.1	I,M,F
Orocit^TM^ (= Vintre™), Oro Agri Inc. Trophy Club, TX	B	0.1–0.8	A
Oroboost^TM^, Oro Agri Inc. Trophy Club, TX	B	0.2–0.8	A
Prev-Am^TM^, Oro Agri Inc. Trophy Club, TX	R	0.4–0.8	I,M,F
Neemix^®^ 4.5, Certis USA, Columbia, MD	B	0.2	I
Kocide^®^ 3000, DuPont^TM^, Wilmington, DE	C	3.2 lbs	F
Cuprofix^®^ Ultra 40, United Phosphorus., King of Prussia, PA	C	3.2 lbs	F
Copper-Count^®^-N, Mineral R&D, Charlotte, NC	C	2 quarts	F
Champ^®^ DP, NuFarm Americas Inc., Burr Ridge, IL	C	4.0 lbs	F

^1^ P = emulsified petroleum-based oil, S = silicon, B = botanical, R = borax, C = copper; ^2^ A = adjuvant, I = insecticide, M = miticide, F = fungicide, PAN Pesticides Database—Pesticide Products, (http://www.pesticideinfo.org/).

*Diaphorina citri* used in these experiments were obtained from the USDA-ARS laboratory colony established during early 2000 at the U.S. Horticultural Research Laboratory, Fort Pierce, FL. Originally collected from citrus, the psyllids have been continuously reared on orange jasmine, *Murraya paniculata* (L.), housed in Plexiglas (0.6 x 0.6 x 0.6 m) or BugDorm-2^®^ cages (MegaView Science Education Services Co., Ltd, Taichung, Taiwan) under the following environmental conditions: 20–28 °C, 40–80% RH and a 14 h light (L):10 h dark (D) photoperiod [32,33]. The original colony has not had field collected psyllids added since establishment.

**Figure 1:**
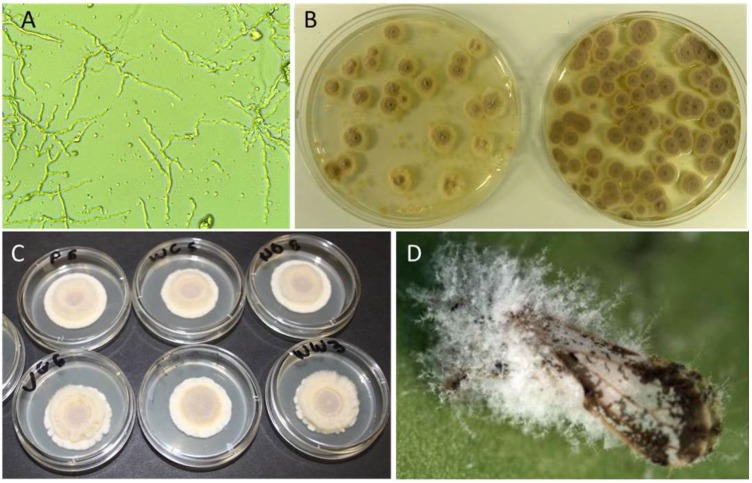
Viability parameters of *Isaria fumosorosea* amended with oil treatments: (**A**) Blastospore germination on potato dextrose agar (PDA), (**B**) Impacts of toxic oils on colony forming units (CFU) (left) compared with controls (right), (**C**) Comparison of radial growth and (**D**) Colonization of adult *D. citri* by fungus and production of conidia.

*Compatibility of* I. fumosorosea *with spray oils and adjuvants* in vitro*:* The compatibility of a blastospore formulation on *I. fumosorosea* Apopka 97 strain with different spray oils or adjuvants was evaluated under sterile conditions in the laboratory. A germination test was conducted by mixing test products at two rates (final concentration 0.5% and 2% v/v) with a suspension of *I. fumosorosea* (final concentration 10^6^ blastospores/mL). Each replicate consisted of 8 mL of test suspension prepared in a sterile 15 mL polypropylene centrifuge vial (Falcon, Colorado Springs, CO). Vials (held horizontally) were maintained on a bench top shaker for 1 hour to simulate mixing in a spray tank. Afterwards, 100 μL of each test suspension was inoculated onto water agar (1.5% w/v, Difco Laboratories, Detroit, MI) in a 9 cm diameter Petri dish and spread over the plate with a glass rod. All plates were sealed with Parafilm^®^ and incubated at 25 °C, 80% RH, 12 h L:12 h D photoperiod. Germination (visible hyphal growth) was assessed after 20 hours from a minimum of 200 blastospores per sample (Figure 1A). Cases of excessive hyphal growth or clumping were not counted. A similar procedure with a lower fungal concentration (10^3^ blastospores/mL) was used to compare the impact of test products (0.5% and 2% v/v) on the number of colony forming units (CFU) produced on potato dextrose agar (PDA) (3.9% w/v, Difco Laboratories) after 4 days (Figure 1B). Tetracycline (0.5% v/v) was added as a bactericide to all plates. 

Two additional experiments estimated the effects of test products on *in vitro* radial growth rates of *I. fumosorosea*. In the first experiment, products were incorporated at two rates (0.5% and 2% v/v) into liquid PDA (3.9% w/v, Difco Laboratories, Detroit, MI) immediately prior to plating in 6 cm diam Petri dishes (10 mL per dish). A 10 μL droplet of a pure *I. fumosorosea* suspension (10^6^ blastospores/mL) was placed in the center of the dish and the resulting colony diameter (mm) was measured at 5, 10 and 15 days post-inoculation. There were three replications per treatment and the study was repeated on five separate occasions. The second study used a slightly lower rate (0.2% v/v) of test materials that were mixed with the fungal suspensions prior to plating, but not incorporated into the PDA media. Replicates consisted of 8 mL of a test suspension prepared in sterile 15 mL centrifuge vials (Falcon, Colorado Springs, CO). Controls for oil treatments were *I. fumosorosea* mixed with sterile water. Vials were vortexed for 60 seconds and a 4 µL droplet of each treatment suspension inoculated on PDA plates (10 cm diam.) with streptomycin (0.5%) added. Plates were sealed with Parafilm^®^ and colony diameter measured at 8, 16, 23 and 30 days post-inoculation (Figure 1C). The test was conducted twice with10 replicates/treatment. Both studies were conducted at 25 °C, 16 h L:16 h D photoperiod.

*Compatibility of* I. fumosorosea *with copper-based fungicides* in vitro*:* The compatibility of a blastospore formulation of *I. fumosorosea* with different copper-based fungicides was evaluated under sterile conditions in the laboratory. Each fungicide was mixed (w/v or v/v) at label rates (Table 1) and incorporated into liquid PDA immediately prior to plating in Petri dishes (15 mm × 100 mm; 10 mL per dish). Four μL of a pure *I. fumosorosea* suspension (10^6^ blastospores/mL) droplet was placed in the center of the dish. Colony diameter (mm) was measured at 20 days post-inoculation. There were 10 replications per treatment and each study was repeated on three separate occasions.

*Compatibility of* I. fumosorosea *with spray oils* in vivo*:* To further investigate compatibility of *I. fumosorosea* and spray oil products, a leaf bioassay was conducted on adult psyllids exposed to afungal suspension with and without test materials. Products were applied to citrus leaves at (10^6^ blastospores/mL) with a Nalgene^®^ aerosol sprayer (Nalge Nunc International, Rochester, NY) as described previously [27]. Controls were leaves sprayed only with water + fungus, and water + oils. Psyllid mortality was assessed daily for 7 days. To investigate whether oils impacted fungal development post-mortem, psyllid cadavers were examined daily at 40X. Individuals were scored according to the fungal development index (FDI) proposed in an earlier publication [27]; *i.e.*, 1.0 (no symptoms); 1.5 = appearance of fungal hyphae through the exoskeleton of the psyllid body; 2.0 = hyphae protruded through head, thorax, wings of host; 2.5 = initial conidia formed on host; 3.0 = fungus has colonized and formed conidia on all sections of the psyllid body (Figure 1D). There were 10 replications per treatment and the study was conducted on two separate occasions.

*Compatibility of* I. fumosorosea *with copper-based fungicide* in vivo: The compatibility of *I. fumosorosea* and copper-based fungicides was assessed using a leaf bioassay similar to that described above. Test materials were applied at label rate to citrus leaves, and allowed to air dry under a fume hood (~2–4 h) prior to the application of the fungus. Adult psyllids were exposed to fungal suspensions with and without fungicides. Psyllid mortality was assessed daily for 12 days and FDI (described above) was used to determine whether fungicides impacted fungal development on the psyllids. There were 10 replications per treatment and both studies were conducted 2–3 times.

*Data analysis:* Treatments from laboratory tests were compared through one and two-way analysis of variance conducted on the data and *post-hoc* means separated where appropriate through Duncan’s multiple range tests at *P* < 0.05. All statistical analyses were conducted using SAS Proc GLM procedures and executed on a WIN_PRO platform (SAS 1999-2001). Insect bioassays were also compared through Kaplan-Meier survival analysis followed by a log rank test (SPSS for Windows v.21 and SAS JMP 8 for Windows 2013). 

## 3. Results

### 3.1. Compatibility of *I. fumosorosea* with Spray Oils and Adjuvants *In Vitro*

Percent germination for the *I. fumosorosea* alone treatments were > 80% for all tests. Analysis of variance (ANOVA) for *in vitro* growth measurements revealed tested citrus products significantly impacted germination (*F*_9, 84_ = 7.1, *P* < 0.0001) with a marginal treatment × rate interaction (*F*_9, 84_ = 2.3, *P* = 0.02). Mean separation tests revealed all citrus products reduced germination at both high (2% v/v) and low (0.5% v/v) rates, while only some reduced numbers of CFU’s at equivalent rates (% v/v) (Table 2). 

**Table 2:** Effect of spray oils and adjuvants at two rates on *in vitro* growth of *I. fumosorosea* with respect to fungus only controls.

Oil/Adjuvant	% Germination		No. of CFU’s	
	0.5% v/v	2% v/v	0.5% v/v	2% v/v
None	100.0a	100.0a	100.0a	100.0a
SuffOil-X	51.2b	29.2bcd	62.3ab	61.3ab
Citrus Wrap	43.7bc	25.9cde	36.4b	35.7bc
Griffin 435	31.0c	28.8bcd	35.7b	45.3bc
PureSpray Green	43.5bc	38.0bc	40.9b	41.8bc
Year-Round	36.2bc	18.9def	41.6b	42.0bc
Sylgard 309	48.4b	45.3b	44.3b	40.8bc
Neem Oil	54.8b	37.2bc	61.1ab	45.0bc
Orocit (=Vintre)	36.7bc	15.3ef	55.0ab	28.3bc

Data are mean of 5 tests (3 replicates per test) and scaled to values obtained in controls in each test. Letters indicate differences (*P* < 0.05, Duncan’s multiple range test). CFU’s = colony forming units.

**Figure 2:**
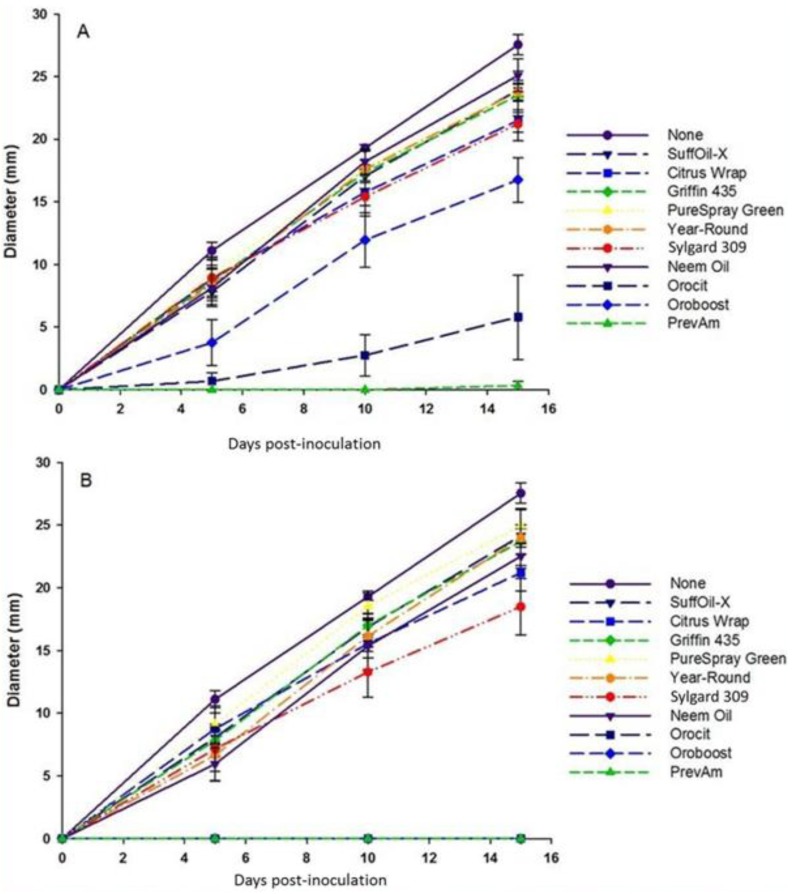
Radial growth estimates for *Isaria fumosorosea* on PDA treated with spray oils and adjuvants at two rates, *i.e.*, A = 0.5% v/v, B = 2% v/v. Data are mean ± SEM of four tests (three replicates per test).

Comparisons of radial growth estimates through repeated measures (RM) ANOVA revealed citrus products incorporated into the media negatively impacted growth over 15 days (*F*_9, 63_ = 67.1, *P* < 0.0001) with a significant treatment × rate interaction (*F*_9, 63_ = 4.2, *P* < 0.001). Comparisons of individual growth rates (*i.e.*, Duncan’s multiple comparison tests in the RM model) revealed that all products significantly impacted growth with the exception of PureSpray Green, SuffOil-X and Griffin 435, and in addition, both the Neem and Year-Round oils did not impact growth at the lower (0.5% v/v) rate (Figure 2). 

Relatively little or no radial growth was observed when citrus oils (Orocit, Oroboost) and borax (PrevAm) were incorporated into PDA media, especially at the 2% v/v rate where growth was completely inhibited (Figure 2). By contrast, in a separate test at the 0.2% v/v rate, radial growth of *I. fumosorosea* suspensions that were pre-mixed with several oils (Orocit, Neemix, Griffin 435 and Oroboost) and plated on pure PDA media that lacked the oils were not subsequently affected compared with controls (RM ANOVA, *F*_4, 5_ = 0.9, *P* = 0.55).

### 3.2. Compatibility of *I. fumosorosea* with Copper-Based Fungicides *In Vitro*

**Figure 3:**
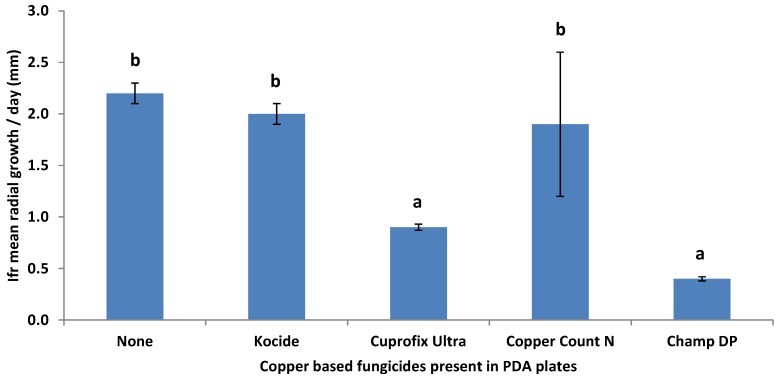
Effect of copper-based fungicides present in PDA plates on the radial growth of *Isaria fumosorosea in vitro* after 20 days post-inoculation. Data are mean ± SEM of two tests (10 replicates per test) and the letters above the bars indicate significance (*P* < 0.05, Duncan's multiple range test).

Percent germination for the *I. fumosorosea* alone was ≥ 83% for all tests. Mean radial growth/day (mm) of *I. fumosorosea* on PDA was impacted by copper-based fungicide treatments (*F*_4, 29_ = 6.81, *P* < 0.0001) (Figure 3). The fungicides that were least compatible with *I. fumosorosea* growth were Champ DP and Cuprofix Ultra, while Kocide and Copper Count N had no apparent impact on fungal growth in this study.

### 3.3. Compatibility of *I. fumosorosea* with Spray Oils *In Vivo*

**Figure 4:**
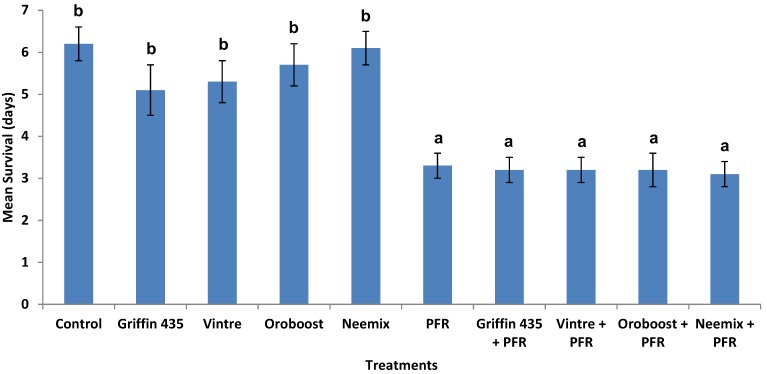
Effect of oils and *Isaria fumosorosea* (PFR) alone and in combination on psyllid survival. Data show survival (days) for *D. citri* exposed to oil (0.2% v/v) treated citrus leaves in the laboratory over 7 days. Data are mean ± SEM of two tests (10 replicates per test) and letters indicate significance (*P* < 0.05, Duncan's multiple range test).

Survival estimates (data pooled across both tests) showed a clear distinction between psyllids exposed to treatments containing *I. fumosorosea* and oil only treatments (Figure 4). Kaplan-Meier analysis (censored at day 7) revealed that oil only treatments survived significantly longer compared with treatments containing *I. fumosorosea*, *i.e.*, average survival 5.7 ± 0.2 days versus 3.7 ± 0.2 days, respectively (log rank *χ*^2^ = 77.0, *P* < 0.0001, df = 1). Additionally, there were no differences between *I. fumosorosea* alone and containing oils, *i.e.*, 3.8 ± 0.3 days versus 3.6 ± 0.4 days, respectively. Comparison of fungal development index (FDI) values on psyllids over 7 days for treatments containing *I. fumosorosea* revealed no impact when various oils were added, compared with oil-free controls (*i.e.*, *F*_1, 5_ = 1.0, *P* = 0.98, in RM ANOVA). Mean natural control (water only) mortality ranged from 0-10% after 6 days and then 0-15% after 7 days.

### 3.4. Compatibility of *I. fumosorosea* with Copper-Based Fungicides *in Vivo*

**Figure 5:**
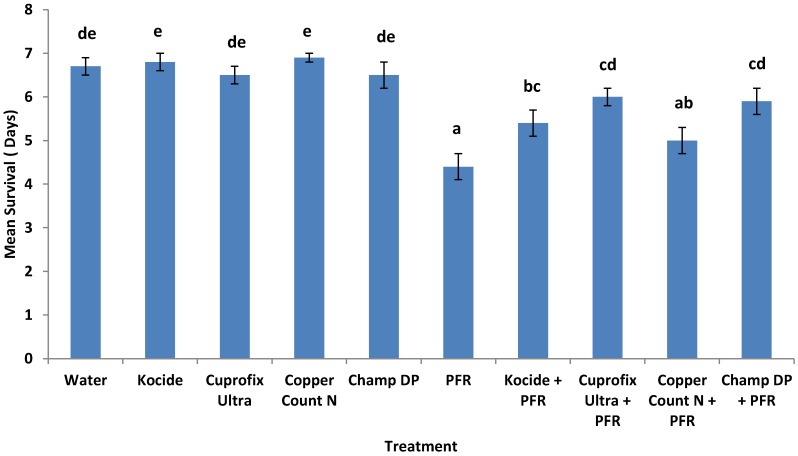
Mean survival (days) for *D. citri* exposed to copper-based fungicide treated citrus leaves prior to application of *Isaria fumosorosea* (PFR) over 7 days. Data are mean ± SEM of three tests (10 replicates per test) and letters indicate significance (*P* < 0.05, Duncan's multiple range test).

**Figure 6:**
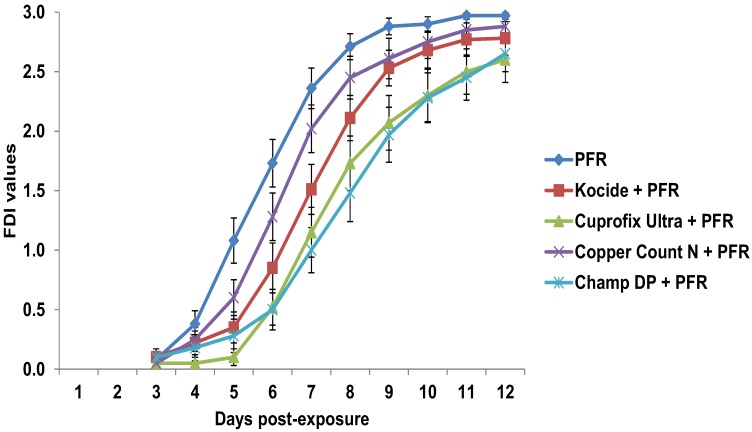
Progression of *Isaria fumosorosea* (PFR) blastospores applied post-application of fungicides on leaves exposed to *D. citri* in laboratory tests assessed using a fungal development index (FDI) [27]. Data are mean ± SEM of three tests (10 replicates per test). Control (water only) mortality was 7% after 7 days and 23% 12 days post-exposure.

Survival estimates of psyllids (data pooled across three tests) indicated an impact of some copper fungicides on fungal performance (Figure 5). Kaplan-Meier analysis (censored at day 7) revealed that psyllids survived longer in *I. fumosorosea* treatments sprayed following copper-based fungicides, *i.e.*, average survival 6.6 ± 0.2 days versus 4.3 ± 0.3 days in *I. fumosorosea* only treatments (log rank *χ*^2^ = 165.8, *P* < 0.0001, df = 9). Mean survival times increased (*F*_4, 29_ = 7.02, *P* < 0.0001) when *I. fumosorosea* was combined with Champ DP, Cuprofix Ultra, and Kocide, but not Copper Count N, compared to the fungus only treatment. Post-mortem development of fungus was affected by copper sprays (*F*_9, 29_ = 137.6, *P* < 0.0001) (Figure 6). Mean natural control (water only) mortality was 3% after 7 days.

## 4. Discussion

Our studies provide information on the compatibility of *I. fumosorosea* with various insecticidal oils adjuvants, and fungicides registered for use in citrus. Germination of blastospores was reduced (or at least delayed) by all tested materials, especially at the higher (2% v/v) application rate for oil when exposed in PDA medium. However, other growth measurements (CFU and radial diameter estimates) were only significantly affected by some of these materials, which may suggest some variation in individual blastospores tolerance to tested products. Overall, *I. fumosorosea* growth *in vitro* were reduced least by petroleum-based materials and most by botanical oils, borax, and some of the copper-based fungicides, suggesting that tank mixing of *I. fumosorosea* with these latter products should be avoided.

However, when psyllids were exposed to fungus/oil residues on leaves *in vivo*, we also observed statistically similar mortality (average survival) compared with the fungus treatment alone. The reasons are unclear, although we hypothesize that some oils may enhance adherence of blastospores to the insect cuticle, overcoming negative impacts on germination. Statistically similar psyllid control was observed in greenhouse tests with *I. fumosorosea* mixed with vegetable-based oil (Addit^®^), compared with either agent sprayed alone [34]. Our findings with *I. fumosorosea* blastospores contrast with previous studies reporting enhanced germination and pathogenicity of conidial formulations of entomopathogenic fungi mixed with many oils and oil-emulsions [35,36,37]. Mechanisms proposed for synergistic activity of oils with entomopathogenic fungi include enhanced adhesiveness of conidia to the lipophilic insect cuticle, disruption (solubilization) of cuticular waxes leading to improved deposition (e.g., by carrying conidia to thin intersegmental membranes) and improved secondary acquisition of fungal inoculum from vegetation or other sprayed surfaces [37,38,39]. A possible explanation for apparent differences in compatibility between blastospore versus aerial conidia relates to their different physiochemical properties. The surfaces of *I. fumosorosea* blastospores are hydrophilic and negatively charged [40]. Thus blastospores are expected to disperse well in water but bind poorly to hydrophobic surfaces. This is in contrast to the typical hydrophobic cell walls of aerial conidia, which adhere rapidly to both hydrophobic and hydrophilic surfaces but may form aggregation in aqueous suspensions [41]. We hypothesize that the thinner and more permeable cell wall of the blastospore may be more readily disrupted by solvent properties of some oils, compared with aerial conidia.

Although petroleum-based (e.g., horticultural mineral) oils are most commonly used in North America, botanical oils (such as neem) are more commonly used in Africa and South America [37]. Several recent studies have investigated the compatibility of entomopathogenic fungi with neem, with variable results. Some studies report that *in vitro* growth of *Beauveria bassiana* (germination, colony formation and sporulation) are negatively affected by emulsifiable neem oils at concentrations as low as 0.5% v/v [42,43,44]. However, other authors report that neem and other plant oils were not toxic to either *I. fumosorosea *or *I. farinosa* [45,46]. There may be variability in susceptibility to oils between fungal strains. Mohan *et al*. [47] tested 30 isolates of *B. bassiana* with a commercial neem formulation (0.3% v/v) and although conidial germination was delayed in all isolates, it was generally not significantly decreased and 23 of the isolates were considered to be compatible. Positive results of neem-based oils combined with various entomopathogenic fungi, *i.e.*, ‘synergism or ‘compatibility’, have also been reported in insect bioassays with caterpillars (*Spodoptera litura*), locusts (*Anacridium melanorhodon*) and mealybugs (*Phenacoccus solenopsis*) [47,48,49]. From these studies, a trend for the improved compatibility of fungi and neem oils in terms of pathogenicity against insects, *versus* direct growth characteristics in media were observed. We report a similar trend in the current study. We also hypothesize that at non-lethal doses, oils might induce physiological stresses to insects that predispose them to infection.

Inhibitory effects of other agrochemicals on the growth and pathogenicity of *I. fumosorosea *and other entomopathogenic fungi has been reported which include several classes of chemical insecticides [50,51,52,53,54,55,56] and systemic fungicides [57]. In the UK, *Lecanicillium muscarium* and *B. bassiana* conidia were found to be compatible with many, but not all, active ingredients (including petroleum oils) used for control of *Bemisia tabaci* [58,59]. Avoiding tank-mixing (*i.e.*, applying materials separately) is a method to reduce incompatibility issues of entomopathogenic fungi and insecticides [51]. Negative impacts of chemical pesticides on entomopathogenic fungi might also be remediated through the combined use of emulsifiable oils. Lopes *et al*. [60] reported that germination rates for unformulated and oil-formulated *Metarhizium anisopliae* conidia exposed to the fungicide carbendazim were reduced by differing amounts (*i.e.*, 77.3 and 12.1%, respectively), compared with fungicide-free controls. It has also been proposed that sub-lethal doses of neonicotinoid insecticides (especially imidacloprid) may suppress insect immunity and enhance the efficiency of entomopathogenic fungi against insect pests [61,62,63,64]. 

Emulsified petroleum oils are recommended for management of psyllids and leafminers in citrus [65]. Commercial formulations of *I. fumosorosea*, such as PFR-97, are currently registered for application in citrus groves. Our data indicate that *I. fumosorosea* has an advantage over oils, *e.g.* psyllids are readily infected by spray residues, whereas oils need to be directly sprayed on the insect to be effective. This aspect may be beneficial in situations where coverage is limited, such as in dense foliage or against psyllids that migrate into the grove shortly after spraying. Dry formulations of *I. fumosorosea* might also be dispersed through autodisseminators for control of psyllids in dooryard citrus trees or plants which are often not sprayed [66,67]. Laboratory insect bioassays also suggest that *I. fumosorosea* might provide control of several additional citrus pests including the *Diaprepes* root weevil adult [30].

A potential limitation for using mycoinsecticides and insecticidal oils together in citrus relates to their lower efficacy compared with some chemical insecticides. In greenhouse tests, *I. fumosorosea* reduced *D. citri* by about 50%, compared with approximately 100% from imidacloprid drenches [34]. This need for more frequent spraying may be perceived negatively by growers. In addition, even under ideal conditions, *I. fumosorosea* takes at least two days to kill *D. citri* [28], and transmission of the citrus greening disease may occur in *D. citri* salivary secretions in as little as 30 minutes of feeding [68]. However, whether the pre-lethal period of infection allows psyllids to spread the HLB pathogen is unclear, since the fungal pathogen dramatically reduces feeding behavior well before killing the host [28]. Further research is needed to assess the ability of fungus-infected psyllids to acquire/transmit the HLB pathogen and disperse it to other plants. 

Copper-based fungicides are commonly used to control diseases caused by plant pathogenic bacteria and fungi in citrus production [31]. We determined that some of these fungicides may also impact the growth of *I. fumosorosea* and hence should be avoided in direct contact (*i.e.*, tank mixing). In addition, *I. fumosorosea* should not be applied on the same day as with copper-based fungicides. Kouassi *et al*. [69] evaluated the compatibility of copper oxide with *B. bassiana*, and suggested that application timing is important for insecticidal activity of the entomopathogenic fungus applied. Copper-based fungicides vary in compatibility, depending on the entomopathogenic fungi being tested, timing of application, and the concentration of the fungicide applied. Demirci *et al*. [57] found that copper oxychloride was compatible and did not inhibit the fungus, *I. farinosa* used against the citrus mealybug. In contrast, D’Alessandro *et al*. [70] discovered that copper oxychloride was incompatible with conidial suspensions of *I. fumosorosea* and significantly reduced mortality of the greenhouse whitefly in comparison to the fungal suspension alone. In our study, we noticed the greatest negative impact on the fungus were from products containing a higher (≥ 40) percent of equivalent metallic copper. In support of this idea, Ali *et al*. [71] reported that increasing concentrations of metallic copper correlated with decreased *in vitro* growth and virulence of *L. muscarium*. On the other hand, it is also possible that fungicides might help *I. fumosorosea* infect and colonize the insects on the phylloplane by eliminating competition with naturally occurring fungi present on the leaves [72]. 

In cases where elimination of greening is no longer paramount, or in organic production where growers accept higher pest levels, the door opens for using more benign materials in an attempt to restore integrated pest management approaches in citrus production. Applying mycoinsecticides and insecticidal oils alone or in combination at critical flushing periods or during dormant periods could help reduce psyllid and other pest populations while having a low impact on beneficial species [73,74,75,76,77]. The effectiveness of the fungus will also depend on distribution of hosts and environmental conditions in the groves. At the time of this writing, the effectiveness of using *I. fumosorosea* in citrus groves largely remains to be established. 

## 4. Conclusions

Our data contributes to an increasing body of work showing that entomopathogenic fungi (mycoinsecticides) may be affected by agrochemicals. A review of such studies reveals various possible interactions, including synergism, antagonism, as well as neutral effects. We note that factors mediating such interactions under field conditions are complex. Further work is needed to optimize the use of mycoinsecticides and insecticidal oils in citrus groves. Compatibilities and timing of applications using *I. fumosorosea* with different fungicides need to be determined prior to being used under field conditions to optimize efficacy of the biopesticide chosen for managing *D. citri*.
